# Cost-effectiveness of Xpert^®^MTB/RIF in the diagnosis of tuberculosis: pragmatic study

**DOI:** 10.1590/0037-8682-0755-2020

**Published:** 2021-02-10

**Authors:** Suely Conceição Alves da Silva, Maria Claudia Vater, Daniela Maria de Paula Ramalho, Isabela Neves de Almeida, Silvana Spíndola de Miranda, Afrânio Kritski

**Affiliations:** 1 Universidade Federal do Rio de Janeiro, Programa Acadêmico de Tuberculose, Rio de Janeiro, RJ, Brasil.; 2 Universidade Federal do Rio de Janeiro, Núcleo de Bioética e Ética Aplicada, Rio de Janeiro, RJ, Brasil.; 3 Universidade Federal de Minas Gerais, Faculdade de Medicina, Laboratório de Pesquisa em Micobactérias, Departamento de Clínica Médica, Belo Horizonte, MG, Brasil.; 4 Universidade Federal de Ouro Preto, Escola de Farmácia, Departamento de Análises Clínicas, Ouro Preto, MG, Brasil

**Keywords:** Multidrug-resistant tuberculosis, Cost-effectiveness, New diagnostics, Xpert MTB/RIF, Innovation

## Abstract

**INTRODUCTION:**

The intensification of research and innovation with the creation of networks of rapid and effective molecular tests as strategies for the end of tuberculosis are essential to avoid late diagnosis and for the eradication of the disease. We aimed to evaluate the cost-effectiveness of Xpert^®^MTB/RIF (Xpert) in the diagnosis of drug-resistant tuberculosis in reference units, in scenarios with and without subsidies, and the respective cost adjustment for today.

**METHODS:**

The analyses were performed considering as criterion of effectiveness, negative culture or clinical improvement in the sixth month of follow-up. The comparison was performed using two diagnostic strategies for the drug susceptibility test (DST), Bactec^TM^MGIT^TM^960 System, *versus* Xpert. The cost effectiveness and incremental cost-effectiveness ratio (ICER) were calculated and dollar-corrected for American inflation (US$ 1.00 = R$ 5,29).

**RESULTS:**

Subsidized Xpert had the lowest cost of US$ 33.48 (R$67,52) and the highest incremental average efficiency (13.57), thus being a dominated analysis. After the inflation was calculated, the mean cost was DST-MGIT=US$ 74.85 (R$ 396,73) and Xpert = US$ 37.33 (R$197,86) with subsidies.

**CONCLUSIONS:**

The Xpert in the diagnosis of TB-DR in these reference units was cost-effective with subsidies. In the absence of a subsidy, Xpert in TB-DR is not characterized as cost effective. This factor reveals the vulnerability of countries dependent on international organizations’ subsidy policies.

## INTRODUCTION

It is estimated that in 2018, there were 10 million new cases of tuberculosis (TB), causing the death of about 1.5 million individuals^1^ and the same year, 72.788 new cases of the disease were reported in Brazil, of which 4.534 died of TB^2^.

Since 2014, the Unified Health System (UHS) incorporated Xpert^®^MTB/RIF (Xpert) in Brazil, after a pilot study carried out in Rio de Janeiro and Manaus^3^. With the incorporation of Xpert, there was no greater access or greater detection of cases of pulmonary TB^4^. Although the federal government has made the molecular test available since 2014, it does not have full coverage in the country. In addition, with the adoption of the molecular test, no drop in the proportion of abandonment or mortality was observed^2^.

The intensification of research and innovation with the creation of networks of rapid and effective molecular tests as strategies for the end of TB is essential to avoid the late diagnosis that hinders the control and eradication of the disease^5^. However, the process of incorporating rapid molecular tests subsidized for countries with high disease burden endorsed by the WHO for TB diagnosis is controversial^6^ ^-^ ^9^, as its incorporation has had a limited impact on health systems, in epidemiological indicators, and the interruption of the transmission chain^7^. This study evaluated the cost effectiveness of Xpert in reference units, in scenarios with and without subsidies and the respective cost adjustment for today, using primary cost data collected during a pragmatic clinical trial.

## METHODS

It is a study of cost effectiveness evaluation of the Xpert test from secondary data on clinical effectiveness available in the literature^10^ and from the analysis of the PROVE IT project database, which allowed the systematization of unpublished cost data from the pragmatic clinical trial.

### Randomized pragmatic clinical trial - PROVE IT

PROVE IT was a study developed in two stages: (1) Baseline, from October 2011 to July 2012, when routine clinical, laboratory, and therapeutic data were collected at each location; (2) Intervention, from August 2012 to May 2013, in which the new molecular tests were incorporated into the routine of each location. All patients were followed for six months by five State and / or National Reference Centers, in four Brazilian states (Rio de Janeiro, Sao Paulo, Ceara and Rio Grande do Sul), for the diagnosis and treatment of DR-TB. For randomization, the Stata software was responsible for allocating units and new technologies (Xpert and Genotype), defining two research branches to ensure each comparative analysis of each study arm: Arm 1: two Health Units used first-line drug susceptibility testing (DST) by Bactec^TM^MGIT^TM^960 System (DST-MGIT) and Xpert; and Arm 2: three units used LPA and MGIT. In this study, only Arm 1 was addressed. 

### Study Population

Eligible patients were aged 18 years or older, presumed DR-TB with one or more of the following conditions: a) failure of previous treatment, b) contacts of DR-TB patients, c) people living with HIV, d) homeless and/or hospitalized, d) and who authorized their participation in the research signing a free and informed consent form. 

### Laboratory Analysis

Tests performed on clinical samples and cultures were performed in reference laboratories that follow the guidelines of the WHO and the National Tuberculosis Control Program of the Ministry of Health of Brazil^11^ ^,^ ^12^.

### Sampling

After the first clinical visit, three moments of monitoring the collection of sputum samples for laboratory and culture analysis were described: 1) screening, 2) follow-up after two months, and 3) six months after the diagnosis was made. Follow-up was defined up to the sixth month, according to the treatment failure criterion for carrying out the DST-MGIT^4^. In this case, the participants were re-evaluated until this moment for the criteria that will be described below in data analysis. Data collection was performed in two reference units for the care of patients with drug-resistant *Mycobacterium tuberculosis* strains; the samples were sent to the Reference Laboratory and the results were released following the routine of each study site.

### Data Analysis

The local research team collected information on the clinical, radiological, and laboratory evolution of the patients using standardized forms, from the clinical-laboratory area and the economic area of the Brazilian Tuberculosis Research Network [Rede Brasileira de Pesquisa em Tuberculose - REDE TB], adapted and used in PROVE IT. The statistical package IBM^®^SPSS for Windows version 20.0 was used for statistical analysis.

The analyses of this study were carried out with the results of the clinical trial^10^: the proportion of patients who received empirical treatment who underwent DST-MGIT (97.7%) and Xpert (45%); the average time between screening and receiving the result of the intervention test by the doctor at DST-MGIT (73 days) and at Xpert (9.10 days); the average time between screening and the beginning of the correct treatment by DST-MGIT (57.7 days) and Xpert (26.8 days). A total of 53 patients were included in the DST-MGIT (n= 36) and Xpert (n= 17), as a criterion of effectiveness: negative sputum result after the sixth month of follow-up (n = 20), of patients who underwent DST-MGIT; negative sputum result after the sixth month of treatment (n = 10), of patients who underwent Xpert; favorable clinical and radiological results of patients who did not have sputum after the sixth month of follow-up of those who underwent DST-MGIT (n = 9) and those who underwent Xpert (n = 6) at the beginning of treatment. 

The follow-up of patients was performed by smear, culture, and DST-MGIT. Xpert was not performed in the follow-up to avoid false positive results.

### Economic Evaluation

 To perform the calculations, the costs related to the monthly expenses of each health unit directly associated with the activities necessary for the laboratory diagnosis of DR-TB were used, considering five cost components: equipment, human resources, supplies, infrastructure, and others (maintenance and loss of damage or misuse). The direct cost of DST-MGIT and Xpert was calculated to assess its impact on the UHS routine, through detailed cost chain modeling. This resulted in the measurement of the values of the following items: ***equipment*** (acquisition of equipment, considering the amortization of capital expenditure), ***human resources*** (salaries of human resources [source: UHS/Ministry of Planning, Development and Management - MPDG] dedicated to the production process related to the diagnostic approach to resistant TB or time spent in the activity and, in the case of non-exclusive dedication, to estimate the professional’s remuneration), ***inputs*** (quantity of material to carry out consumption tests per year). Of the indirect costs, ***infrastructure*** (need to adapt the physical area, air-conditioned environment, electrical and third-party equipment, water, electricity, energy, and security) corresponded to the percentage spent on each item carried out by the set of activities involving TB in each health unit. The cost calculations of the new Xpert technology, since equipment and cartridges were not purchased at market price, considered the values “with subsidy” and “without subsidy” from the Foundation for Innovative New Diagnostics (FIND)^10^. The technique known as “mean cost” was the sum of the monthly expenses of all items used in the exam, divided by the number of exams performed in a month^13^.

For the cost-effectiveness analysis, a comparison of the two diagnostic strategies DST-MGIT versus Xpert for DR-TB was performed, considering the effectiveness of negative culture or clinical improvement in the sixth month of follow-up.

To assess the impact of variations in both effectiveness and cost components, in the sensitivity analysis, we consider the confidence interval (IC 95%) presented in the change of the initial conduct and the variation of -50% to + 25% in the final value of the cost in each technology on the incremental cost-effectiveness ratio (ICER). ICER was defined as the result of the division between the difference in cost of new technologies and the difference in their effect [ICER = (C_1_ - C_2_)/(E_1_ - E_2_)]. where C1 = alternative cost A, C2 = alternative cost B, E1 = effectiveness A, and E2 = effectiveness B^14^.

The proportion of discount used was 5%, according to the recommendations of the Methodological Guidelines for Studies of Economic Evaluation of Technologies for the Ministry of Health^14^.

The incremental cost of Xpert was calculated in two different scenarios: the first considering the value of equipment and cartridges at market prices (without subsidy), and the second with values subsidized by FIND/Diagnostic.

The inflation corrections in the country of origin and importing country were calculated according to the exchange rate variation of the commercial dollar in the period from 2012 to 2020, considering the inflation history for both countries (USA and Brazil) of the software reference for converting historical currencies and exchange rates, FXtop^15^. 

### Sensitivity analysis of incremental effectiveness

Sensitivity analysis was performed using the maximum and minimum values found in the clinical trial for both Xpert and DST-MGIT in the field of effectiveness. The sensitivity analysis was calculated as follows: in the first case, the average value of the DST-MGIT (80.55%) was fixed, varying the XPERT to its maximum (99) and minimum (71) effectiveness, and in the case of Xpert (94.12%), varying the DST-MGIT in its maximum effectiveness (91) and at least (65).

Regarding cost, two variables were used, with subsidy and without subsidy, dividing the values by the effectiveness according to the minimum and maximum variations, as described in the previous section.

This study used the variation in effectiveness as secondary data of the clinical study performed with the SPSS statistical package, version 20.0.

### Ethical Consideration

The study was approved by the Ethics Advisory Group at the International Union Against Tuberculosis on May 10, 2011, EAG 11/11, and by the National Ethics Committee on September 29, 2011, register-1657, 2500.115789 / 2011-94.

## RESULTS

### Cost-effectiveness Analysis

The cost of DST-MGIT calculated using the mean cost method was US$ 67.13. There is a large weight of inputs, representing 80.44% of the total cost (Table 1 and  Supplement Material).

**TABLE 1:** Mean cost of the Drug Susceptibility Test MGIT and Xpert^®^MTB/RIF.

	DST-MGIT			Xpert with subsidy			Xpert without subsidy		
Cost Items	MC(R$)	MC(US$)	%	MC(R$)	MC(US$)	%	MC(R$)	MC(US$)	%
Equipment	1,43	0.71	1.06	1,96	0.97	2.90	12,33	6.12	3.49
Human Resources	24,21	11.99	17.88	29,70	14.73	43.99	29,70	14.74	8.40
Inputs	108,91	54.01	80.44	34,0	16.86	50.36	309,47	153.46	87.58
Infrastructure	0,84	0.42	0.62	1,86	0.92	2.75	1,86	0.92	0.53
Total	135,39	67.13	100	67,52	33.48	100	353,36	175.24	100

**Legend:** MC: Mean Cost; *American dollar on September 11, 2012, conversion value US$1.00 = R$ 2,0165; Xpert: Xpert^®^MTB/RIF.

The mean cost of Xpert without subsidy and with subsidy was US$ 175.24 and US$ 33.48, respectively. In the composition of the total cost, the percentage of inputs was 87.58% without subsidy and 50.36% with subsidy (Table 1 and  Supplement Material).

In Table **2**, the cost effectiveness by Xpert technology, with subsidy had a value of US$ 0.36 in relation to negative culture in the sixth month and the clinical improvement; the cost effectiveness without subsidy had a value of US$ 1.86 ( Supplement Material).

**TABLE 2:** Cost effectiveness and Incremental Cost-Effectiveness Ratio.

Diagnostic Test	Mean Cost	Effectiveness	Cost	Incremental	Incremental	ICER
	(US$)	(negative culture	Effectiveness	Effectiveness	Cost	(US$)
		and clinical improvement)	(US$)		(US$)	
DST-MGIT	67.13	80.55	0.83	-	-	-
Xpert with subsidy	33.48	94.12	0.36	13.57	dominated	dominated
Xpert without subsidy	175.24	94.12	1.86	13.57	108.11	7.97

**Legend:** American dollar conversion value US$1.00 = R$ 2,0165 on September 11, 2012. **ICER:** incremental cost-effectiveness ratio; **DST:** drug susceptibility test; **MGIT:** Bactec^TM^MGIT^TM^960 System; **Xpert:** Xpert^®^MTB/RIF; **ICER:** Incremental Cost-Effectiveness Ratio.

The incremental difference cost Xpert in relation to the o DST-MGIT was a strong dominance of Xpert, when calculating the value of the equipment and cartridges of the market price with subsidy, and the incremental effectiveness was 13.57, which is a dominated analysis (Table 2 and  Supplement Material).

The dollar correction for American inflation^15^ of the diagnostic tests shows in June 2020 the values of US$ 74.85 (DST-MGIT), US$ 37.33 (Xpert with subsidy) and US$ 195.38 (Xpert without subsidy). Likewise, due to the correction of inflation, the incremental and ICER costs assume the values of US$ 120.54 and US$ 8.89, respectively (Table 3 and  Supplement Material).

**TABLE 3:** Cost effectiveness and ICER dollar-corrected for American inflation*.

Diagnostic Test	Mean Cost	Effectiveness**	Cost	Incremental	Incremental Cost	ICER
	(US$)		Effectiveness (US$)	Effectiveness	(US$)	(US$)
DST-MGIT	74.85	80.55	0.93	13.57	-	-
Xpert with subsidy	37.33	94.12	0.40		dominated	dominated
Xpert without subsidy	195.38	94.12	2.07		12.54	8.89

**Legend:** *American inflation = 11.49% on period of 11/09/2012 at 02/06/2020 in https://fxtop.com/pt/calculadora-de-inflacao.php; American dollar conversion value US$1.00 = R$ 5,2993 in June, 2 of 2020. **Negative culture and clinical improvement. **ICER:** Incremental Cost-Effectiveness Ratio; **DST**: Drug Susceptibility Test; **MGIT:** Bactec^TM^MGIT^TM^960 System; **Xpert:** Xpert^®^MTB/RIF.

In the local currency, the MGIT amount went from R$ 135,39 to R$ 396,73 and Xpert with a subsidy from R$ 67,52 to R$ 197,86 and Xpert without a subsidy from R$ 353,36 to R$ 1.035,48. Likewise, the incremental and ICER costs the Xpert without a subsidy from R$ 218,00 to R$ 638,80 and R$ 16,07 to R$ 47,10, respectively (Table 4 and  Supplement Material). 

**TABLE 4:** Exchange variation - import R$/Brazil.

	Test 2012	American	Test 2020	Exchange Rate	Real Value 2020
	(R$/BRL)	Inflation	(R$/BRL)	Variation	(R$/BRL)
DTS-MGIT Cost	135,39	11.49%	150,94	162.84%	396,73
Xpert with subsidy	67,52		75,28		197,86
Xpert without subsidy	353,36		393,96		1.035,48
Xpert Incremental cost without subsidy	218,00		243,04		638,80
Xpert Cost Effectiveness Incremental without subsidy	16,07		17,92		47,10

**Legend:** 11.49% = on period of 11/09/2012 at 02/06/2020 in https://fxtop.com/pt/calculadora-de-inflacao.php; American dollar on September 11, 2012, conversion value US$1.00 = R$ 2,0165 and R$ 5,2993 in 02.06.2020. DST: Drug Susceptibility Test; MGIT: Bactec^TM^MGIT^TM^960 System; Xpert: Xpert^®^MTB/RIF; BRL: Brazil.

### Sensitivity analysis of incremental effectiveness

The analysis of the increment in three situations: in the first, varying Xpert effectiveness within the values of 71% (minimum) and 99% (maximum), the negative value after six months was maintained at 80.55% for DST-MGIT; in the second, 94.12% negativity for Xpert was maintained and DST-MGIT negativity varied between 65% and 91%, according to the values found. A value for negative culture or clinical improvement of Xpert of 71% was obtained, characterizing a scenario without incremental effectiveness; in the third, the variation was in cost when the value of Xpert without subsidy was used, with an average value of US$175.24 (Table 5 and  Supplement Material).

**TABLE 5:** Incremental effectiveness by variation Maximum and Minimum Xpert with subsidies and DST-MGIT.

Xpert with Subsidies				
	Xpert	Xpert (variation) and DST-MGIT (fixed) 80.55%		
Maximum	99.00	18.45		
Average	94.12	13.57 Incremental effectiveness		
Minimum	71.00	Negative (-9.55)		
	DST-MGIT	DST-MGIT (variation) and Xpert (fixed) 94.12%		
Maximum	91.00	3.12		
Average	80.55	13.57 Incremental effectiveness		
Minimum	65.00	29.12		
		
**Xpert**	**Incremental cost**	**Test’s effectiveness**		**ICER**
**Without subsidy**	**Xpert without subsidy less**	**and Standard Deviation**		
	**DST-MGIT**			
US$ 175.24	US$108.11	Maximum 99.00	Xpert (variation) and DST-MGIT (fixed) 80.55%	US$ 5.85
		Average 94.12		US$ 7.97
		Minimum 71.00		dominated
		Maximum 91.00	DST-MGIT (variation) and Xpert (fixed) 94.12%	US$ 34.65
		Average 80.55		US$ 7.97
		Minimum 65.00		US$ 3.71

**Legend: DST:** Drug Susceptibility Test; **MGIT:** Bactec^TM^MGIT^TM^960 System; **Xpert:** Xpert^®^MTB/RIF; **ICER:** incremental cost-effectiveness ratio.

The values found when negative in the sixth month or clinical improvement of Xpert, assumes a minimum value of 71%; therefore, it is a dominated analysis. In the case of the cost of Xpert without subsidy, the ICER was US$ 7.97. When considering the 91% effectiveness for DTS-MGIT, ICER was US$ 34.65 for six months’ negative results and clinical improvement (Table 5 and  Supplement Material).

## DISCUSSION

Xpert in subsidized conditions was more cost effective than DST-MGIT for incorporating the new technology in the routine diagnosis of DR-TB in the Brazilian UHS. In addition to the dependency condition (subsidy), the cost-effectiveness analysis showed that the cartridge used by the Xpert machine had the highest percentage in calculating the total cost of this technology. The percentage of the cartridge cost was 50.36% of the final cost in the test with subsidy and 87.58% without subsidy. This input, being imported, reinforces the vulnerability in a situation of exchange rate depreciation, which can bring economic limitations for the incorporation of technology into the national health system. Contingent factors of market relations and/or changes in the position of a developing country^16^ can lead to the main cost component (cartridge) of the test to assume the market value, ceasing to be cost-effective for the criterion of negativity in the sixth month and clinical improvement of DR-TB patients in the studied sites.

Xpert was cost effective when compared to DST-MGIT when analyzing sensitivity variation for both incremental effectiveness and incremental cost. In addition, it presented significant advantages with regard to the increase in TB detection and its satisfactory accuracy; however, there are controversies regarding its implementation in order to support decision making^8^ ^,^ ^17^. An investigation^9^ showed that the benefits expected from the rapid detection of Xpert were not reflected in the improvement of the patient throughout the treatment; consequently, the costs did not decrease, even with subsidized incentive policies in South Africa. Similar results can be found in a study^7^ analyzing the limited impact of Xpert and its gaps in the ability to produce and maintain an effect for the patient, also observed^17^ when there was no significant difference in the proportions of cure among patients allocated to the Xpert group and the control group or, as shown in another study^18^ that, despite having found excellent accuracy, suggests additional studies aimed at the evaluation of the Xpert impact on the patient.

In a recent meta-analysis carried out by the Collaborative Group for the Meta-Analysis of Data of Individual Patients in the treatment of MDR TB, the “surprising” discovery of the lack of benefit of several drugs commonly prescribed for MDR TB treatment was highlighted^19^.

In order to address the problem of the increase in DR/MDR/XDR-TB, the race for molecular tests that are faster and more accurate in the diagnosis has been the object of permanent research in the world^5^ ^,^ ^20^ ^,^ ^21^. The Xpert system is an example of the global expansion of philanthropy characterized by the incorporation of technological innovation products in the healthcare market. The development of Xpert for TB control was subsidized by the Gates Foundation's 15 million USD investment in the Cepheid Company. FIND monitored contract expenditures for the development of the Xpert cartridge for US$7.23 million and US$1 million for the calibration kit required by the test^22^. The remaining US$6.8 million of the grant went to clinical trials and project management that enabled the partnership among industry, academia and governments in several countries^22^. 

From the point of view of the data found in this study, this kind of “philanthropic episteme” became necessary and important because it has the strength to show with transparency the birth of the innovation and research and development (R&D) activities of the studied technology, and also to estimate the variability of the intersection point that involves the incorporation of subsidized technologies in low-and middle-income and dependent countries. Among the countries in South America that incorporated Xpert, Brazil continues to be the main customer in the acquisition of machines and cartridges^23^, as it is the largest public continental health system. In 2018, the Ministry of Health took the strategic decision to purchase more diagnostic equipment from the GeneXpert system, totaling the coverage of 135 municipalities with 249 machines^2^ ^,^ ^4^.

The clinical decision made by doctors in prescribing tuberculosis treatment (empirical treatment) before test results was less for the group of patients at Xpert (65% Xpert and 97% DST-MGIT), which is in line with the statement of the potential of rapid tests to reduce empirical treatment and better control DR-TB. However, the finding of 65% of prescriptions for treatment of probability when having a test that presents sensitivity 82% to 93% and specificity 96% to 100%, seems to reveal that under routine of the studied places the time between the laboratory result and the doctor's receipt of this does not correspond to the standard established by the technology provider^24^ ^,^ ^25^. 

An important contribution of this study and consistent in different cases^12^ is that clinical trials in the routine of the units have significance for impact assessment and the incorporation of new diagnostic tests, due to the potential to demonstrate organizational problems in the local health system that make it difficult to receive the treatment properly and in the shortest time possible.

The dependence on new technology and exchange variation are factors that proved to be central in this cost analysis, which was also demonstrated in a study^26^ stating that subsidies should be considered in decision making in addition to other factors that, from a future perspective, they impact the real cost of a new test for dependent countries^26^. When considering American inflation, between the years 2012 and 2020^15^, on the total cost of each diagnostic test, an increase in the ICER was observed from US$ 7.97 to US$ 8.89. These results, corrected by the US inflation accumulated in the period in the conversion of the current exchange rate to the importing country, generate the cost adjustment and make the new test no longer cost-effective, in relation to the other, which shows the “subsidized condition” as a vulnerability in incorporation in dependent countries: Xpert with subsidy R$ 67,52 - US$ 33.48 to R$ 197,86 - US$ 37.33, Xpert without subsidy R$ 353,36 - US$ 175.24 to R$ 1.035,48 -US$ 195.38 and DST-MGIT R$ 135,39-US$ 67.13 to R$ 396,73 - US$ 74.85. 

These results call attention to the fact that added to the factor (inflation of the country of origin that produces the technology), it is also necessary to take into account the variation in the exchange rate of the American currency in the importing country (Brazil). Thus, the devaluation of the currency (R$) has an effect on the increase in the exchange rate of the American currency, which results in the need for a larger amount of reais to purchase the same dollar unit. In this scenario, the budgetary impact in relation to the import of equipment and/or inputs can be catastrophic for the health system, even in a subsidized situation, reinforcing that subsidies must be taken into account in decision-making in peripheral countries. 

The confidence that evidence is capable of inspiring for decision making, central to the evaluation of new diagnostic tests in TB^21^, finds the best investigative model in pragmatic clinical trials, as these occur in the routine of services, with patients who really depend on adequate treatment. Therefore, it is essential to consider that in the analysis of the accuracy and usefulness of a new technology, it must be subject to applicability in the “real life” of the local health system, in order to answer the questions of essential and sustainable adjustments in the treatment of DR-TB.

It was evident that the implementation of diagnostic tests in the routine showed a statistical difference in the favorable outcomes of the treatment of DR-TB (p = 0.04) in patients whose clinical samples were submitted to Xpert compared to those submitted to DST-MGIT in the analyzed period. However, the difference between the cost-effectiveness results is reversed if the subsidy is withdrawn and, due to the cost adjustment for the current year, the subsidized test is not cost effective. The definition of prioritization criteria, considering the possible scenarios and the implicit risks of subsidized costs, to justify the decision to incorporate the new test in the UHS were not investigated in this study.

However, in the evidence-based deliberative process proposed in other studies^25^ ^,^ ^27^, there is a complexity in this path because the definition is the result of a political process loaded with value and divergences. Therefore, the cost evaluation will need to be weighed by the relative importance of the criteria for choosing those involved in the deliberation process. Furthermore, it has been highlighted that the dominant use of cost-effectiveness information for decision-making may not be the fairest choice for universal health systems. Finally, it is argued that the assessment of health technologies needs to make explicit the evidence-based deliberative process to facilitate learning among stakeholders.

In the decision-making for the subsidized incorporation of a new diagnostic test in the UHS, the analysis of the cost impact without Xpert subsidy does not seem to have prevailed. To contribute to the eradication of the disease, this choice could be weighted by the improvement in epidemiological indicators, which did not occur in this Brazilian scenario^4^ ^,^ ^28^ ^,^ ^29^.

This study had limitations regarding the small number of patients included in the clinical intervention stage and not having followed up the patients until the end of MDR-TB treatment, although it is representative of the reality of the routines of each participating health unit. Another limitation, since the completion of this study to the present day, there are technical variations in laboratory activity that have not been analyzed. Due to the clinical and laboratory peculiarities of each location, generalization of results is not allowed. 

In conclusion, in the context of the data presented here, Xpert in the diagnosis of TB-DR in these reference units was cost effective. When corrected by the US $ inflation accumulated in the period in the conversion of the current exchange rate to the importing country, generate the cost adjustment and make the new test no longer cost-effective. For decision making by UHS managers, there is still a great challenge due to the complexity of the Brazilian health system, which requires more pragmatic clinical trials. In addition, in cases where the technology has a large weight of imported items, attention should be paid to factors such as the local exchange rate and inflation in the country where the technology is produced, which should be included in the economic assessment of the incorporation of new methods of diagnosis in resistant tuberculosis. 
