# Supplementary material for: Cost-effectiveness of Xpert®MTB/RIF in the diagnosis of tuberculosis: pragmatic study
Source: Rev Soc Bras Med Trop. 2021 Feb 10;54:e07552020. doi: 10.1590/0037-8682-0755-2020 (PMC7891564; doi:10.1590/0037-8682-0755-2020)
Supplement: Supplementary file 1 [file 1678-9849-rsbmt-54-e07552020-suppl1.pdf]

Supplementary Material

1. Mean Cost Analisys Calculation Memory

Xpert with subsidy in National Referral Center in State of Rio de Janeiro (n =160)

Activities	Amount	Unit cost (US$)	Total cost (US$)	Mean Cost (US$)
**Equipaments**				US$ 1.23
Xpert IV	1	17.000.00	17.000.00	0.89
Computer	1	495.91	495.91	0.05
Stable and uninterrupted electrical supply	1	495.91	495.91	0.05
Printer	1	198.36	198.36	0.02
Shipment	1	4.156.73	4.156.73	0.22
**Human Resources**				US$ 18.28
Laboratory Technician	3	717.42	2.152.26	13.45
Administrative assistent	1	772.28	772.28	4.83
**Inputs**				US$ 16.86
Cartridge	1	16.86	16.86	16.86
**Infrastructure**				US$ 1.22
Light	Monthly bill	17.356.81	17.356.81	0.22
Water	Monthly bill	59.509.05	59.509.05	0.74
Safety	Monthly bill	12.382.90	12.382.90	0.15
Cleaning	Monthly bill	8.678.40	8.678.40	0.11
**Total**				US$ **37.59**

Legend: Xpert: Xpert®MTB/RIF

Xpert with subsidy in National Referral Center in State of Ceará (n=275)

Activities	Amount	Unit cost (US$)	Total cost (US$)	Mean Cost (US$)
**Equipaments**				US$ 0.71
Xpert IV	1	17.000.00	17.000.00	0.52
Computer	1	495.91	495.91	0.03
Stable and uninterrupted electrical supply	1	495.91	495.91	0.03
Printer	1	198.36	198.36	0.01
Shipment	1	4.156.73	4.156.73	0.12
**Human Resources**				US$ 11.18
Laboratory Technician	5	495.91	2.479.54	9.02
Administrative assistent	1	595.09	595.09	2.16
**Inputs**				US$ 16.86
Cartridge	1	16.86	16.86	16.86
**Infrastructure**				US$ 0.62
Light	Monthly bill	50.501.58	50.501.58	0.37
Water	Monthly bill	10.676.12	10.676.12	0.08
Safety	Monthly bill	8.628.39	8.628.39	0.06
Cleaning	Monthly bill	15.473.66	15.473.66	0.11
**Total**				US$ **29.37**

Legend: Xpert: Xpert®MTB/RIF

Xpert with subsidy: total Mean Cost (US$)			
Activities	Mean Cost (US$): A	Mean Cost (US$): B	Mean Cost (US$)
**Equipaments**	1.23	0.71	**0.97**
**Human Resources**	18.28	11.18	**14.73**
**Inputs**	16.86	16.86	**16.86**
**Infrastructure**	1.22	0.62	**0.92**
**Total**	37.59	29.37	**33.48**

Legend: A = National Referral Centers in State of Rio de Janeiro; B = National Referral Center in State of Ceará; Xpert: Xpert®MTB/RIF

Xpert without Subsidy in National Referral Center of Rio de Janeiro (n=160)				
**Activities**	**Amount**	**Unit cost (US$)**	**Total cost (US$)**	**Mean Cost (US$)**
**Equipaments**				US$ 7.73
Xpert IV	1	139.463.41	139.463.41	7.26
Computer	1	1.487.73	1.487.73	0.15
Stable and uninterrupted electrical supply	1	743.86	743.86	0.08
Printer	1	198.36	198.36	0.02
Shipment	1	4.156.73	4.156.73	0.22
**Human Resources**				US$ 18.28
Laboratory Technician	3	717.42	2.152.26	13.45
Administrative assistent	1	772.28	772.28	4.83
**Inputs**				US$ 153.47
Cartridge	1	153.47	153.47	153.47
**Infrastructure**				US$ 1.22
Light	Monthly bill	17.356.81	17.356.81	0.22
Water	Monthly bill	59.509.05	59.509.05	0.74
Safety	Monthly bill	12.382.90	12.382.90	0.15
Cleaning	Monthly bill	8.678.40	8.678.40	0.11
**Total**				**US$ 180.71**

Legend: Xpert: Xpert®MTB/RIF

Xpert without Subsidy in National Referral Center in State of Ceará (n=275)				
**Activities**	**Amount**	**Unit cost (US$)**	**Total cost (US$)**	**Mean Cost (US$)**
**Equipaments**				US$ 4.50
Xpert IV	1	139.463.41	139.463.41	4.23
Computer	1	1.487.73	1.487.73	0.09
Stable and uninterrupted electrical supply	1	743.86	743.86	0.05
Printer	1	198.36	198.36	0.01
Shipment	1	4.156.73	4.156.73	0.13
**Human Resources**				US$ 11.18
Laboratory Technician	5	495.91	2.479.54	9.02
Administrative assistent	1	595.09	595.09	2.16
**Inputs**				US$ 153.47
Cartridge	1	153.47	153.47	153.47
**Infrastructure**				US$ 0.62
Light	Monthly bill	50.501.58	50.501.58	0.37
Water	Monthly bill	10.676.12	10.676.12	0.08
Safety	Monthly bill	8.628.39	8.628.39	0.06
Cleaning	Monthly bill	15.473.66	15.473.66	0.11
**Total**				**US$ 169.77**

Legend: Xpert: Xpert®MTB/RIF

Xpert without Subsidy: total Mean Cost (US$)			
**Activities**	**Mean Cost (US$): A**	**Mean Cost (US$): B**	**Mean Cost (US$)**
**Equipaments**	7.73	4.50	**6.11**
**Human Resources**	18.28	11.18	**14.74**
**Inputs**	153.47	153.47	**153.47**
**Infrastructure**	1.22	0.62	**0.92**
**Total**	180.71	169.77	**175.24**

Legend: A = National Referral Center in State of Rio de Janeiro; B = National Referral Center in State of Ceará; Xpert: Xpert®MTB/RIF

DST-MGIT in National Referral Center in State of Rio de Janeiro (n=325)				
**Activities**	**Amount**	**Unit cost (US$)**	**Total cost (US$)**	**Mean Cost (US$)**
**Equipaments**				US$ 0.92
Biological Safety Cabin	1	7.438.63	7.438.63	0.10
Centrifuge	1	981.90	981.90	0.01
Shaker	1	210.36	210.36	0.00
Bactec MGIT 960	1	61.988.59	61.988.59	0.79
Pipettor	1	47.61	47.61	0.00
Telefone	1	14.33	14.33	0.00
Sink	1	99.18	99.18	0.00
Hand sanitizer	1	28.76	28.76	0.00
Garbage basket	1	9.87	9.87	0.00
Computer	1	743.86	743.86	0.01
Stopwatch	1	10.90	10.90	0.00
Metal tray	1	24.80	24.80	0.00
Centrifuge tube rack 30 mL or 50 mL	1	13.89	13.89	0.00
Pasteur pipettes (2mL and 5mL)	3	0.31	0.94	0.00
Glass or metal container for material disposal	1	0.50	0.50	0.00
Polypropylene tray for incubating seeded tubes	1	34.71	34.71	0.00
**Human Resources**				US$ 16.95
Biochemical	1	1.173.63	1.173.63	3.61
Laboratory technician	5	717.42	3.587.11	10.84
Administrative agent	1	772.28	772.28	2.37
Safety	Monthly bill	12.382.90	12.382.90	0.08
Cleaning	Monthly bill	8.678.40	8.678.40	0.05
**Inputs**				US$ 54.01
4% NaOH solution	5.00	0.00	0.01	0.01
Neutralizing Solution	5.00	0.50	2.48	2.48
70% Alcohol Solution (mL)	5.00	0.00	0.01	0.01
5% Phenol solution.	5.00	0.50	2.48	2.48
Phosphate Buffer Solution pH 6.8	5.00	0.08	0.40	0.40
Sterile distilled water	5.00	2.95	14.75	14.75
Sterile Saline Solution	5.00	0.02	0.10	0.10
2.9% Sodium Citrate Solution	5.00	0.50	2.48	2.48
Absorbent paper (roll)	0.05	1.26	0.06	0.06
Polypropylene centrifuge tubes	1.00	0.50	0.50	0.50
Sterile gauze in pieces (piece)	2.00	0.00	0.00	0.00
5% Phenol Solution Container	1.00	0.50	0.50	0.50
Plastic container for the material to be autoclaved and discarded.	1.00	0.50	0.50	0.50
Tubes with Liquid Medium - 7H9 + Antibiotic + Supplement (udd)	4.00	7.27	29.06	29.06
Smear slides (unit)	2.00	0.04	0.07	0.07
Autoclavable plastic bag for disposal	0.03	0.50	0.02	0.02
Mask	0.03	3.02	0.10	0.10
Disposable apron	0.17	0.05	0.01	0.01
Disposable Glove	0.17	0.01	0.00	0.00
PH tape and Petri dish or small test tube	1.00	0.50	0.50	0.50

Continued				
**Infrastructure**				US$ 0.62
Telefone	Monthly bill	24.299.53	24.299.53	0.15
Light	Monthly bill	17.356.81	17.356.81	0.11
Water	Monthly bill	59.509.05	59.509.05	0.37
**Total**				**US$ 72.50**

Legend: DST: Drug Susceptibility Test; MGIT: Bactec™MGIT™960 System; Xpert: Xpert®MTB/RIF

DST-MGIT in National Referral Center in State of Ceará (n=600)				
**Activities**	**Amount**	**Unit cost (US$)**	**Total cost (US$)**	**Mean Cost (US$)**
**Equipaments**				US$ 0.50
Biological Safety Cabin	1	7.438.63	7.438.63	0.05
Centrifuge	1	981.90	981.90	0.01
Shaker	1	210.36	210.36	0.00
Bactec MGIT 960	1	61.988.59	61.988.59	0.43
Pipettor	1	47.61	47.61	0.00
Telefone	1	14.33	14.33	0.00
Sink	1	99.18	99.18	0.00
Hand sanitizer	1	28.76	28.76	0.00
Garbage basket	1	9.87	9.87	0.00
Computer	1	743.86	743.86	0.01
Stopwatch	1	10.90	10.90	0.00
Metal tray	1	24.80	24.80	0.00
Centrifuge tube rack 30 mL or 50 mL	1	13.89	13.89	0.00
Pasteur pipettes (2mL and 5mL)	3	0.31	0.94	0.00
Glass or metal container for material disposal	1	0.50	0.50	0.00
Polypropylene tray for incubating seeded tubes	1	34.71	34.71	0.00
**Human Resources**				US$ 7.02
Biochemical	1	991.82	991.82	1.82
Laboratory technician	5	495.91	2.479.54	4.13
Administrative agent	1	595.09	595.09	0.99
Safety	Monthly bill	8.628.39	8.628.39	0.03
Cleaning	Monthly bill	15.473.66	15.473.66	0.05
**Inputs**				US$ 54.01
4% NaOH solution	5.00	0.00	0.01	0.01
Neutralizing Solution	5.00	0.50	2.48	2.48
70% Alcohol Solution (mL)	5.00	0.00	0.01	0.01
5% Phenol solution.	5.00	0.50	2.48	2.48
Phosphate Buffer Solution pH 6.8	5.00	0.08	0.40	0.40
Sterile distilled water	5.00	2.95	14.75	14.75
Sterile Saline Solution	5.00	0.02	0.10	0.10
2.9% Sodium Citrate Solution	5.00	0.50	2.48	2.48
Absorbent paper (roll)	0.05	1.26	0.06	0.06
Polypropylene centrifuge tubes	1.00	0.50	0.50	0.50
Sterile gauze in pieces (piece)	2.00	0.00	0.00	0.00
5% Phenol Solution Container	1.00	0.50	0.50	0.50

Plastic container for the material to be autoclaved and discarded.	1.00	0.50	0.50	0.50
Tubes with Liquid Medium - 7H9 + Antibiotic + Supplement (udd)	4.00	7.27	29.06	29.06
Smear slides (unit)	2.00	0.04	0.07	0.07
Autoclavable plastic bag for disposal	0.03	0.50	0.02	0.02
Mask	0.03	3.02	0.10	0.10
Disposable apron	0.17	0.05	0.01	0.01
Disposable Glove	0.17	0.01	0.00	0.00
PH tape and Petri dish or small test tube	1.00	0.50	0.50	0.50
**Infrastructure**				US$ 0.23
Telefone	Monthly bill	1.787.35	1.787.35	0.02
Light	Monthly bill	50.501.58	50.501.58	0.16
Water	Monthly bill	10.676.12	10.676.12	0.05
**Total**				**US$ 61.76**

Legend: DST: Drug Susceptibility Test; MGIT: Bactec™MGITTM960 System; Xpert: Xpert®MTB/RIF

	DST-MGIT: total Mean Cost (US$)		
**Activities**	**Mean Cost (US$): A**	**Mean Cost (US$): B**	**Mean Cost (US$)**
**Equipaments**	0.92	0.50	**0.71**
**Human Resources**	16.95	7.02	**11.99**
**Inputs**	54.01	54.01	**54.01**
**Infrastructure**	0.62	0.23	**0.42**
**Total**	72.50	61.76	**67.13**

Legend: A = National Referral Center in State of Rio de Janeiro; B = National Referral Center in states of Ceará; DST: Drug Susceptibility Test; MGIT: Bactec™MGIT™960 System; Xpert: Xpert®MTB/RIF

2. Cost Effectiveness Analisys Calculation Memory

Xpert and DST-MGIT

Arm1* Negativity_6ºmonth_lab_and_clinic Cross tabulation

Arm 1	Negativity_6ºmonth_lab_and_clinic		Total exams	Effectiveness
	Negative	Positive		
Xpert	16	1	17	= 94.12%
DST-MGIT	29	7	36	= 80.55%
Total	45	8	53	

Effectiveness = Tt exams / Tt negativity 6º month
Xpert = 17/16 = 94.12%
DST-MGIT = 36/29 = 80.55%

Incremental Effectiveness = Effectiveness Xpert – Effectiveness DST-MGIT
Incremental Effectiveness = [94.12] – [80.55] = 13.57

Cost effectiveness and Incremental Cost-Effectiveness Ratio (ICER) – (Manuscript Table 2)

Diagnostic Test	Mean Cost (US$)	Effectiveness (negative culture and clinical improvement)	Cost Effectiveness (US$)	Incremental Effectiveness	Incremental Cost (US$)	ICER (US$)
DST-MGIT	67.13	80.55	0.83	-	-	-
Xpert with subsidy	33.48	94.12	0.36	13.57	dominat	dominat
Xpert without subsidy	175.24	94.12	1.86	13.57	108.11	7.97

Legend: DST: Drug Susceptibility Test; MGIT: Bactec™MGIT™960 System; Xpert: Xpert®MTB/RIF

Cost Effectiveness = Mean Cost / Effectiveness

Incremental Effectiveness = Effectiveness Xpert – Effectiveness DST-MGIT

Incremental Cost = Mean Cost Xpert – Mean Cost DST-MGIT

ICER Cost = (Mean Cost Xpert – Mean Cost DST-MGIT) / Incremental Effectiveness

3. Sensitivity Analysis Calculation Memory (Manuscript Table 5)

Xpert With Subsidies		
	Xpert	Xpert (variation) and DST-MGIT (fixed) 80.55%
Maximum	99.00	(Maximun - Average) + Incremental effectiveness
Average	94.12	Incremental effectiveness (Manuscript Table 2)
Minimum	71.00	(Average – Minimum) - Incremental effectiveness
	DST-MGIT	DST-MGIT (variation) and Xpert (fixed) 94.12%
Maximum	91.00	(Maximun - Average) + Incremental effectiveness
Average	80.55	Incremental effectiveness – (Manuscript Table 2)
Minimum	65.00	- (Average – Minimum) - Incremental effectiveness

Xpert Without subsidy	Incremental cost Xpert without subsidy less DST-MGIT	Test's effectiveness and Standard Desviation		ICER
Mean Cost (US$) (Manuscript Table 2)	Incremental Cost (US$) (Manuscript Table 2)	Maximum 99.00	Xpert (variation) and DST-MGIT (fixed) 80.55%	Incremental cost/(Maximun - Average) + Incremental effectiveness (xpert)
		Average 94.12		ICER (US$) (Manuscript Table 2)
		Minimum 71.00		Incremental cost/(Average – Minimum) + Incremental effectiveness (xpert)
		Maximum 91.00	DST-MGIT (variation) and Xpert (fixed) 94.12%	Incremental cost/(Maximun - Average) + Incremental effectiveness (DST-MGIT)
		Average 80.55		ICER (US$) (Manuscript Table 2)
		Minimum 65.00		Incremental cost/(Average – Minimum) + Incremental effectiveness (DST-MGIT)

Legend: DST: Drug Susceptibility Test; MGIT: Bactec™MGIT™960 System; Xpert: Xpert®MTB/RIF; ICER: Incremental Cost-Effectiveness Ratio
